# Rubber Dam and Its Application

**Published:** 1873-05

**Authors:** 


*Rubber Dam and its Application.*—In the *Dental Advertiser* Dr. G. C. Daboll explains his method of applying the "rubber dam" as follows :

To young operators, or to older ones, who have had little experience in the application of the rubber dam, many difficulties are presented which seem almost insurmountable, especially when they are obliged to operate without an assistant. This is owing almost entirely to its peculiar nature, which renders it so difficult to keep in its position until it can be secured. It is a simple matter to make a hole in the rubber and stretch it over the tooth, but it is quite another to hold it there until it can be forced into a safe position. Having been through all the phases of this difficulty, and arrived at a fair degree of success, I herewith give some of my experience, hoping it may be not without profit to some who are still having trouble. One should be provided, first, with a strong thread or twine that will bear strain enough to carry it between the closest and most firmly set teeth. In my own practice I have found saddlers' thread, thoroughly waxed, to answer an admirable purpose. Each ligature should be from twelve to twenty inches in length, and it is well to have several cut off and prepared before commencing the operation, in case of emergency. The first step taken is to pass the ligature through all the spaces between the teeth over which it is proposed to put the dam, and in almost every operation it is advisable to put it over from two to four teeth. The object of carrying the ligature through first, is to ascertain how close the spaces are, and to carry any little points of calculus or other deposit that may be on the approximal surfaces, and also to get a correct idea of the difficulty one has to contend with.

Taking, for instance, the second left inferior molar, which, in the average mouth, would be rather difficult, and supposing the operator to be alone, the *modus operandi* would be to prepare the rubber for four teeth, the bicuspids and molars, leaving a margin wide enough from the holes, so that when the mouth is open the edges will come well up over the upper lip and the corner of the mouth. Now carrying the first hole over the first bicuspid, well down on the lingual and buccal surfaces, direct the patient to place the index finger of the left hand on the buccal surface of the tooth, with an injunction to hold it steadily and firmly ; after that is in position repeat the operation with the index finger of the right hand for the lingual surface. With a little care most patients readily get the idea and but little difficulty will be experienced in making them understand what is wanted. Now carry the ligature firmly down between the canine and the bicuspid, laying it across the crown of the bicuspid and sliding it to the space, to be sure that a double thickness of the rubber is not carried down. Now draw this ligature through until just the end is left, and cut it off, the end remaining in most cases being amply sufficient to secure the position. Repeat this process on the second bicuspid and first molar, which will ordinarily be easier ; the real trouble commencing when we reach the second molar. The ligature being carried down between the molars—there being no dens sapientia—it should be left there ; then carrying the last hole over the molar, hold the rubber with the index finger of the left hand, and with the foil carriers, or an instrument, carry the ligature around the distal surface, bringing both ends together, and direct the patient to hold them firmly, having taken care that it is sufficiently well on the tooth to prevent its being pulled off ; now, with a point bent at an angle sufficient for the purpose, make sure that the ligature is carried down below the swell of the crown, and direct the patient, after getting the instrument in position, to hold it there while the knot is tied. After a trial or two it will be found easy to tie the knot on the distal surface, making it still more secure than if tied on the buccal surface. This represents as difficult a case as one ordinarily meets with, much depending on the mouth and the patient, as regards the ease with which the molars can be reached ; much depends, also, on the patience and perseverence, to say nothing of the ingenuity, that is required of the operator. Many little points are to be observed in the management of the rubber. The care required in making the holes, in spacing them so that when carried down between the teeth it shall fill the space closely at the margin of the gum—otherwise the stretch will render it so narrow as to cause a leak. Whatever may be the result of a first trial in any case, don't let a failure discourage you, but avail yourself of the knowledge born of experience, and try again. It will pay, for operations that were formerly considered impracticable are, by this simple appliance, rendered as easy as the simplest.

